# Immunohistochemical Detection of Human Papillomavirus 16 E7 Oncoprotein in Cervical Lesions

**DOI:** 10.7150/jca.60554

**Published:** 2021-10-20

**Authors:** Jiang Zhu, Lingjing Chen, Qiaoli Zheng, Rui Han, Xianzhen Chen, Qiang Zhou, Hao Cheng

**Affiliations:** 1 Department of Dermatology, Sir Run Run Shaw Hospital, School of Medicine, Zhejiang University, Hangzhou 310016, Zhejiang Province, China.; 2 Department of Dermatology, Hangzhou Children's Hospital, Hangzhou 310014, Zhejiang Province, China.

**Keywords:** Human papillomavirus (HPV), E7 protein, Polyclonal antibody, Immunohistochemistry, Cervical cancer

## Abstract

Almost all cervical cancer is associated with persistent infection of high-risk (hr) human papillomavirus (HPV) like HPV16. The E7 oncoprotein encoded by hrHPV plays a crucial role in carcinogenesis. The present study aimed to establish a reliable protocol of immunohistochemistry stains to detect HPV16 E7 protein in formalin-fixed and paraffin-embedded tissue specimens of various cervical lesions. Firstly, the HPV16 E7 gene was inserted into a prokaryotic expression vector pGEX-4T2. Then the recombinant plasmid pGEX-4T2-(HPV16-E7) was transformed into *Escherichia coli* JM109. The fusion protein containing a GST tag was purified, and New Zealand white rabbits were immunized to produce the HPV16 E7 polyclonal antibody. The anti-HPV16 E7 antibody was verified by western blotting and immunofluorescence stains, and applied in 182 HPV16 DNA-positive cervical tissue specimens and matched 36 HPV DNA-negative specimens by immunohistochemistry. Furthermore, A positive correlation between HPV16 E7 protein expression and malignancy grade was observed. But there is no relationship between HPV16 E7 protein expression and tumor sizes, tumor differentiation, lymph node metastasis, International Federation of Gynecology and Obstetrics (FIGO) stage, or lymphovascular space invasion in cervical cancer. These findings provide a basis for further research focusing on the role of HPV E7 protein in various HPV-related diseases.

## Introduction

Cervical cancer is the fourth most common cancer and the fourth leading cause of cancer death in women worldwide. According to the World Health Organization's global cancer observatory, there were an estimated 570,000 new cases and 311,000 deaths in 2018 worldwide. Besides, the incidence rate was 2-fold higher, and the mortality rate was 3-fold higher in low/medium Human Development Index (HDI) regions compared with high/very-high HDI regions 1, contributing to a massive public health burden, especially in low/medium HDI regions. The high-risk human papillomavirus (hrHPV) is a virtually necessary cause of cervical cancer, which could be detected in 99.7% of cervical cancer cases 2. Persistent infection of hrHPV in the cervical epithelium was considered as a pivotal figure to cause cervical carcinogenesis 3, in which HPV16 and HPV18 had been classified as group 1 carcinogens by the IARC Monographs. It has been widely proved that early screening, diagnosis and treatment of HPV infection was an effective way to prevent cervical cancer.

The HPV genome consists of approximately 7,900 base pairs that can be divided into three regions: an early (E) region, a late (L) region, and a non-coding long control region containing regulatory elements for viral DNA replication and transcription. The L genes encode structural proteins. The E region contains six open reading frames (E1, E2, E4, E5, E6, E7) that are mainly for regulatory functions engaged in genome persistence 4, DNA replication 5, lytic cycle activation 6, and immune evasion 7. The E6 and E7 proteins as oncoproteins could interact with cell cycle checkpoints mechanisms by inactivating tumor suppressor proteins p53 and retinoblastoma protein to promote viral replication 8. Besides, E6 and E7 proteins could interfere with the NF-κB pathway and interferon to escape immune detection 9. Therefore, the E7 protein is one of the significant factors of HPV-associated carcinogenesis with markedly upregulated during transformation.

HPV genotyping analysis was widely used in clinical practice and provided great prevention against invasive cervical carcinomas 10. But it could not distinguish from transient or persistent infection limits its predictive value. Therefore, oncoprotein E7 consistently expressed in HPV‑transformed cervical cells could be a better diagnostic marker. The aim of this study was the expression of the HPV16 E7 protein *in vitro*, preparation of polyclonal anti-HPV16 E7 antibody, and its clinical application in various cervical lesions to investigate the relationship between HPV16 E7 protein expression and multiply clinical features.

## Materials and Methods

### Patients, Animals and Cell lines

This study was approved by the Ethics Committee of the Sir Run Run Shaw Hospital, College of Medicine, Zhejiang University and conducted with written informed consents of all participants. Formalin-fixed and paraffin-embedded cervical tissue specimens and their corresponding patient data were obtained from the Departments of Obstetrics and Gynecology, Sir Run Run Shaw Hospital, School of Medicine, Zhejiang University between 2009 and 2016 by cervical biopsy or hysterectomy. Patient data including tumor size, tumor differentiation grade, lymphovascular invasion, and lymph node metastasis was reviewed respectively. All patients were staged according to the International Federation of Gynecology and Obstetrics stage system, histologically classified and graded according to the WHO classification criteria, cytologically classified according to the Bethesda classification by two experienced pathologists. The inclusion criteria were described as follows: (1) polymerase chain reaction (PCR) proved HPV16 DNA-positive cervical lesions, (2) pathologists get consensus with histological classification, grade and cytological classification. The exclusion criteria were described as follows: (1) patients received chemotherapy, radiotherapy or immunotherapy before specimen collection. (2) tumor recurrence.

All animal procedures were performed in accordance with animal use protocols approved by Zhejiang Chinese Medical University Animal Care and Use Committee and Zhejiang University Animal Care and Use Committee. New Zealand white rabbits (3 months old, weighing approximately 2.5 kg) were supplied by the Laboratory Animal Research Center of Zhejiang Chinese Medical University (certificate number SYXK, Zhejiang, 2013-0184) to raise polyclonal antibodies. Human embryonic kidney (HEK) 293T cell line and human cervical squamous cell carcinoma SiHa cell line were cultured in Dulbeccos modified Eagles Medium (DMEM) supplemented with 4.5g/L glucose and 10% fetal bovine serum (FBS, Sijiqing, China).

### Plasmid Construction and Protein Purification of HPV16 E7

The HPV16 E7 gene was amplified by PCR from HPV16 genomic DNA (ATCC, USA). The forward primer (5′ CCGGGATCCATGCATGGAGATACACCTAC 3′) and the reverse primer (5′ CCGGAATTCTTATGGTTTCTGAGAACAGATG 3′) containing *Bam*HI and *Eco*RI restriction enzyme sites were synthesized by Qingke Biological Technology Co., Ltd. (Zhejiang, China). The PCR product was cleaved with *Bam*HI and *Eco*RI, purified and ligated to the *Bam*HI- and *Eco*RI-digested pGEX-4T2 vector. pGEX-4T2-(HPV16-E7) encodes the HPV16 E7 protein containing a GST open reading frame. The *Escherichia coli* strain JM109 (Takara, Japan) was used as the host. The resultant plasmid was further confirmed to be correct by double restriction enzyme digestion and sequencing (Qingke Biological Technology Co., Ltd., Zhejiang, China).

The strains harboring HPV16 E7 were grown in LB medium containing 100μg/ml ampicillin shaken at 250 rpm and 37°C until the optical density (OD) 600nm reached 0.6-0.8. Isopropyl β-D thiogalactopyranoside with a final concentration of 0.2mM was added, and the culture was incubated at 26°C for 4-6 hours. Cells were harvested by centrifugation, resuspended in PBS, and sonicated on ice. The homogenate was centrifuged at 2,000 × g for 5min, both supernatants and pellet fractions were collected. The fusion protein was purified by affinity chromatography with glutathione-Sepharose 4B beads (Life, USA), the GST-tag was removed by thrombin (Life, USA), the purified protein was dialysed against PBS overnight and stored at -80°C.

### Production and Purification of Anti-HPV16 E7 Polyclonal Antibodies

To produce anti-HPV16 E7 polyclonal antibodies, New Zealand white rabbits were immunized by subcutaneous injection of about 500 μg HPV16 E7 (in 0.8ml) mixed with an equal volume of Freund's complete adjuvant (Sigma-Aldrich, USA). Booster injections were applied as described above but using Freund's incomplete adjuvant at 10-day intervals three times. The quality of the antibodies in sera was monitored by indirect enzyme-linked immunosorbent assay (ELISA), which was repeated daily until a threshold was determined (1:100,000).

For ELISA, as the antigen for ELISA, purified HPV16 E7 proteins were diluted to a final concentration of 50 μg/ml in 0.05 M carbonate buffer solution (pH 9.6) at 37°C for one hour and at 4°C overnight. After washing with PBS containing 0.05% Tween 20 and blocking with 200 μL 5% milk at 37℃ for 2 hours, 100 μL anti-HPV16 E7 polyclonal antibody (1:100,000 diluted) was used as the primary antibody, and HRP-conjugated goat anti-rabbit antibody was used as the secondary antibody. TMB Solution was used as the color substrate and terminated with TMB Stop Solution, the OD450 was detected. Serums with the OD450 values higher than that of control serums by 2.1-fold were defined as positive.

Another 500 μg HPV16 E7 without adjuvant was injected ten days later. Rabbit sera were collected five days after the last immunization. The Protein G Agarose (Life Technology) was applied to purify polyclonal immunoglobulin according to the manufacturer's protocol. The serum was stored at -20°C in aliquots.

### Western blotting analysis and Immunofluorescent

For western blotting, cell lysates and protein samples were subjected to SDS-PAGE and transferred to PVDF membrane (Bio-Rad, USA). Membranes were blocked in PBS-T containing 5% w/w non-fat milk (BD Difco^TM^, USA). The rabbit polyclonal antibodies raised against HPV16 E7 were used as the primary antibodies. The peroxidase-conjugated anti-rabbit immunoglobulin G (IgG; diluted 1:5000 in PBS; Beyotime) was used as the secondary antibodies. Protein was detected with Enhanced Chemiluminescence (ECL) western blotting substrate (Millipore, USA) through a chemiluminescence imaging system (Fujifilm, USA).

For immunofluorescence, 293T cells and SiHa cells were fixed in 4% paraformaldehyde (Bio-Rad, USA) and permeabilized with 0.1% Triton X-100 (Solarbio, China). Anti-HPV16 E7 polyclonal antibody was used as the primary antibody and Alexa Fluor 488-conjugated donkey anti-rabbit IgG (1:500; Beyotime) was used as the secondary antibody. DAPI (Beyotime) was applied to stain the nucleus and stainings were observed under an immunofluorescence microscope (Olympus, USA).

### HPV16 E7 Immunohistochemistry

Immunohistochemistry stains were performed as follows. Formalin-fixed paraffin-embedded tissue sections (8 μm) were deparaffinized in xylene and rehydrated in graded ethanol. Heat-induced antigen retrieval was performed using citrate buffer (pH 6.0). Slides were treated with 0.3% hydrogen peroxide for 30 minutes to quench endogenous peroxidase activity, blocked with PBS containing 10% normal goat serum at room temperature for 30 minutes. Rabbit anti-HPV16 E7 polyclonal antibody (1:400) was used as the primary antibody and the horseradish peroxidase-conjugated goat anti-rabbit IgG antibody (1:1000) was applied as the secondary antibody. Slides were then incubated with DAB (3, 3'-diaminobenzidine) for 5 minutes, counterstained with hematoxylin for 5 seconds, visualized using a microscope (Carl Zeiss, Germany).

Five random high power fields (400×) were observed to score the staining intensities and positive cell percentages. The staining intensities were quantified as follows: 0 (no staining); 1 (light brown); 2 (brown); 3 (dark brown). The positive cell percentages were graded as follows: 0 (0%); 1 (1-25%); 2 (26-50%); 3 (51-75%); 4 (76-100%). The immunoreactive score (values, 0-12) was finally obtained by calculating the product of the staining intensity (0-3) and the positive cell percentage (0-4). According to the immunoreactive score, the staining results were defined as follows: negative, score 0-1; low expression, score 2-3; moderate expression, score 4-5; high expression, score ≥ 6. The results were evaluated by two independent pathologists.

### Statistical analysis

Spearman's rank correlation test was used for comparison between groups. All statistical analyses were performed using the SPSS 20.0 statistical software (SPSS Inc., Chicago, IL, USA). *P* value < 0.05 were considered statistically significant.

## Results

### Expression of HPV16 E7 protein

**Figure 1:**
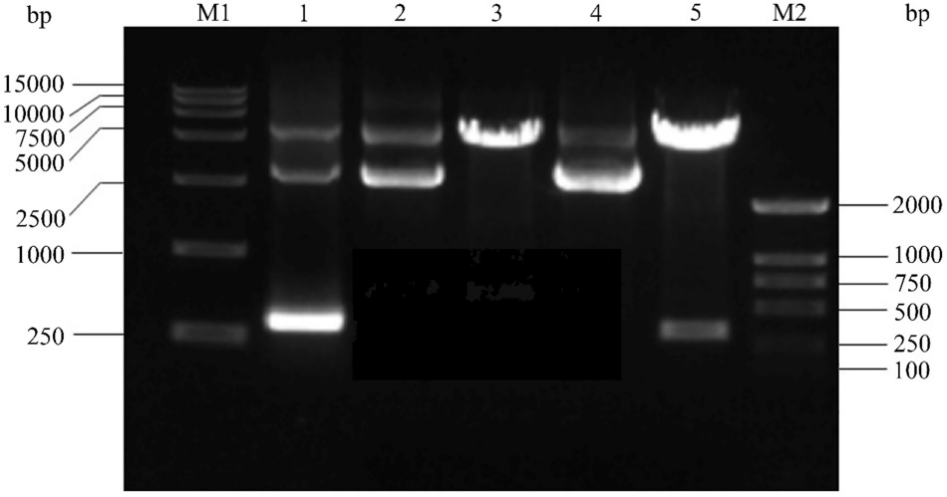
The construction of the HPV16 E7 prokaryotic expression vector. M1: DNA DL15000; Lane 1: Amplification product of HPV16 E7 by polymerase chain reaction (PCR) from HPV16 purified plasmid DNA; Lane 2: pGEX-4T2 vector; Lane 3: pGEX-4T2 vector was digested with the restriction enzymes of *EcoR* I and *BamH* I; Lane 4: recombinant plasmid pGEX-4T2-(HPV16-E7); Lane 5: recombinant plasmid was digested with the restriction enzymes of *EcoR* I and *BamH* I; M2: DNA DL2000. HPV, Human papillomavirus.

**Figure 2:**
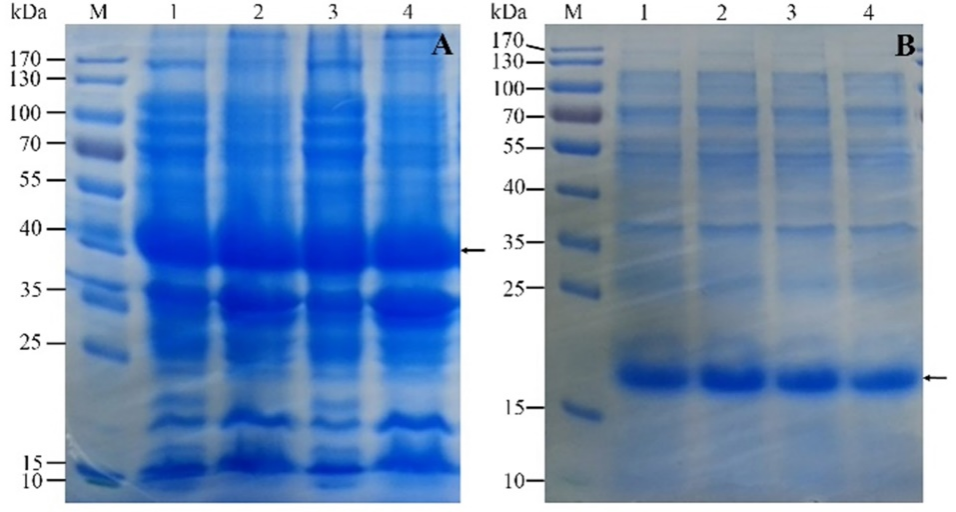
The expression and purification of recombinant HPV16 E7 protein. (A) Sodium salt-polyacrylamide gel electrophoresis (SDS-PAGE) analysis of the expression of the protein in JM109. M: protein marker; Lane 1 and 3: the supernatant of cells; Lane 2 and 4: the pellet of broken cells. Arrow indicates GST-(HPV16-E7) protein. (B) The SDS-PAGE analysis of the purification of the protein. M: protein marker; Lane 1 and 2: recombinant protein after removed GST-tag; Lane 3 and 4: the purified protein after dialyzed overnight. Arrow indicates HPV16 E7 protein.

Firstly, The HPV16 E7 gene was inserted into the pGEX-4T2 vector. The recombinant pGEX-4T2-(HPV16-E7) vectors were identified by gene sequencing and double enzyme digested with *BamH* I and *EcoR* I (Figure 1). Secondly, the vectors were transformed into *Escherichia coli* JM109 and induced with IPTG. Thirdly, the expressed HPV16 E7 fusion protein containing GST-tag was purified by Glutathione-Sepharose 4B beads, removed GST-tag by thrombin, and dialyzed overnight. Both fusion protein and purified protein were separated by 10% SDS-PAGE and visualized by Coomassie blue staining (Figure 2). It indicated that the molecular weight of the HPV16 E7 fusion protein was about 37 kDa and the purified protein was about 18 kDa.

### Expression of HPV16 E7 polyclonal antibody

**Figure 3:**
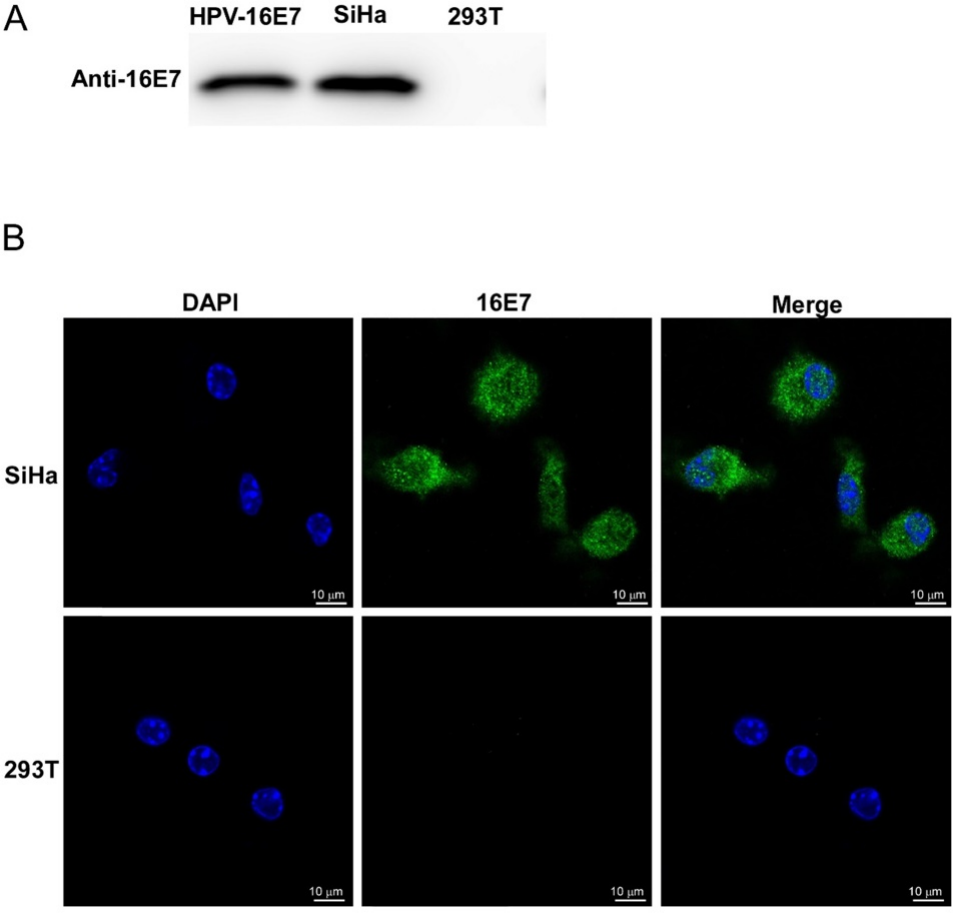
The specificity of the purified anti-HPV16 E7 polyclonal antibody. (A) Western blot analysis. HPV16 E7: purified HPV16 E7 protein; 293T: 293T cells lysates; SiHa: SiHa cells lysates. (B) Immunofluorescence stains. Photomicrographs showed the distribution of immunoreactive HPV16 E7 protein (green) and DAPI-stained nucleus (blue) in SiHa cells and 293T cells.

The HPV16 E7 polyclonal antibody was obtained from the rabbits that were immunized five times with purified HPV16 E7 protein. Rabbit serum antibody concentrate was monitored by ELISA and purified by the Protein G Agarose. The specificity of the purified anti-HPV16 E7 polyclonal antibody was verified by both western blotting and immunofluorescent stains (Figure 3). SiHa cells were known to continually expressing HPV16 E7 protein endogenously, and 293T cells were negative of HPV16 E7 protein. As shown in Figure 3, HPV16 E7 protein was detected by the homemade anti-HPV16 E7 polyclonal antibody in SiHa cell lysates but not in 293T cell lysates. Besides, our previous study proved that there is no cross-reaction of the polyclonal antibody between different HPV genotypes 11.

### Expression of HPV16 E7 protein in cervical lesions

**Figure 4:**
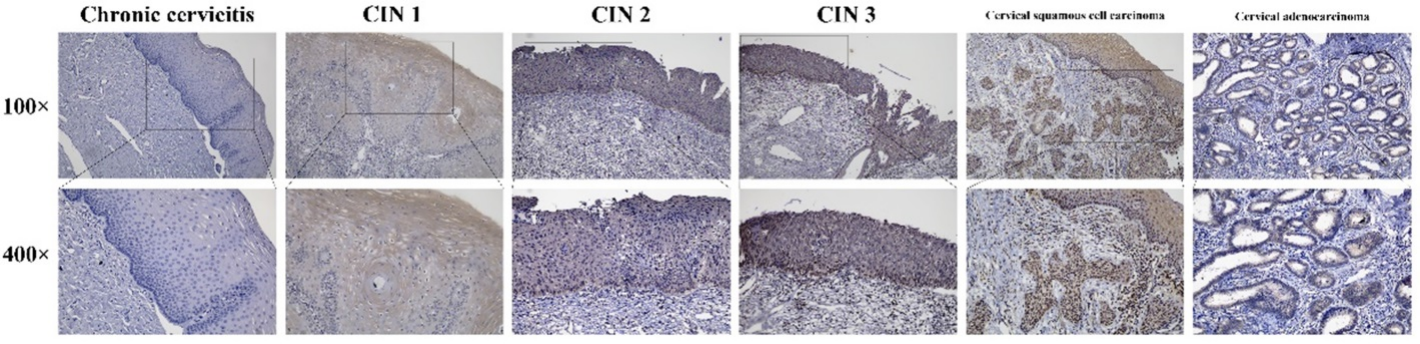
Immunohistochemistry stains of the HPV16 E7 protein in various cervical lesions. The purified HPV16 E7 antibody was used as a primary antibody in chronic cervicitis, cervical intraepithelial neoplasia (CIN) 1, CIN 2, CIN 3, cervical squamous cell carcinoma, and cervical adenocarcinoma tissue specimens. The brown color indicates positive stain.

**Figure 5:**
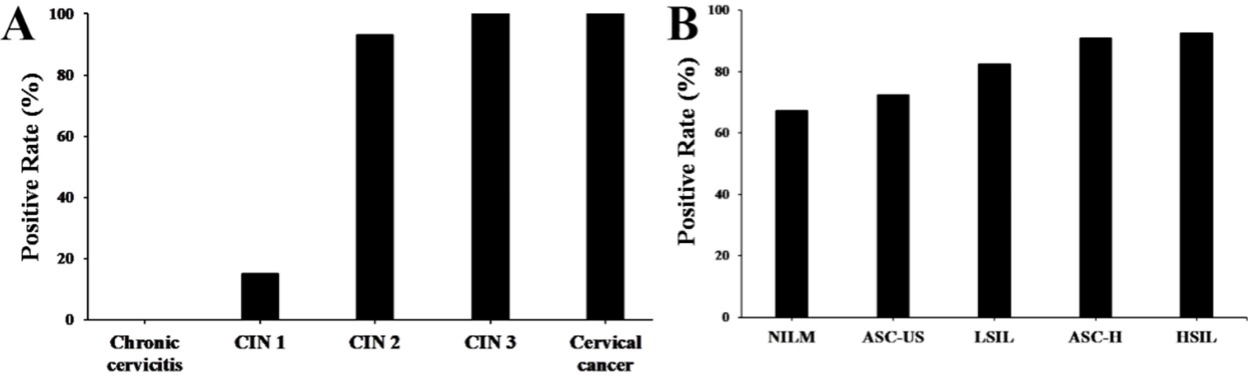
The positive rate of HPV16 E7 protein in various cervical lesions. (A) The positive rate of HPV16 E7 protein in cervical lesions with various histological grades according to the FIGO staging systems. (B) The positive rate of HPV16 E7 protein in cervical lesions with different cytological grades according to the Bethesda systems.

Immunohistochemistry stains of HPV16 E7 protein were applied in a total amount of 182 specimens of PCR proved HPV16 DNA-positive cervical lesions and 36 matched HPV16 DNA-negative chronic cervicitis tissue specimens (Figure 4). It also indicated that the HPV16 E7 protein was localized in the nucleus. As shown in Figure 5, the positive rate of HPV16 E7 protein expression was elevated with increasing either histological or cytological malignancy grade. It also showed a positive correlation between HPV16 E7 protein expression positive rates and histological grade (r=0.675, *p* < 0.01), HPV16 E7 protein expression levels and histological grade (r=0.756, *p* < 0.01), and HPV16 E7 protein expression positive rates with cytological malignancy grade (r=1, *p* < 0.01) (Table 1 and 2). Since all 182 samples were PCR proved HPV16 DNA-positive, the positive coincidence rate of HPV16 E7 protein immunohistochemistry stain compared to HPV genotyping analysis in cervical intraepithelial neoplasia 1 (CIN1), CIN2, CIN3, and cervical cancer was equal to HPV16 E7 protein expression positive rates as 15%, 93%, 100%, and 100%. There is a positive correlation between HPV16 E7 protein immunohistochemistry stain and HPV genotyping analysis in positive coincidence rate (r=0.675, *p* < 0.01). The negative coincidence rate was 100% accordingly. However, for the different tumor sizes, tumor differentiation, lymph node metastasis, International Federation of Gynecology and Obstetrics (FIGO) stage, or lymphovascular space invasion in 58 cases of cervical cancer, there is no relationship between these subgroups in HPV16 E7 protein expression levels (Table 3) (*p* > 0.05, respectively).

## Discussion

The HPV infection is associated with a broad spectrum of diseases that range from self-limiting skin warts to life-threatening cancers. The hr-HPV has been confirmed with close links to invasive cervical cancer, especially HPV16 and 18. Moreover, E7 protein encoded by hr-HPV types plays a crucial role in carcinogenesis by the following mechanism: Initially, E7 oncoproteins might down-regulate TLR-9, a fundamental role in pathogen recognition and innate immune activation, to evade the immune response and cause persistent infection 12. Consequently, E7 oncoproteins could degrade tumor suppressor proteins p53 and retinoblastoma protein and inhibit cyclin dependent kinase inhibitors (p21, p27, p16) to disrupt cell cycle checkpoint control, which results in host cells proliferation without DNA damage repaired. Besides, E7 could bind to STING, an adaptor protein downstream of the intracellular DNA sensor via the LXCXE motif, to suppress a post-infection immune response 13. E7 could also bind to and augment c-Myc-mediated transcription activity, which Myc is inarguably the most potent oncogene in the human cell, to induce uncontrolled cell growth and proliferation 14. Additionally, E7 could cause DNA damage and chromosomal aberrations due to replication stress. Silencing of E7 revealed a significant decrease in DNA damage 15. Furthermore, E7 could induce abnormal centrosome synthesis, which is another mechanism of genomic instability in malignant tumors, leading to aneuploidy 16. In the epigenetic regulation, E7 could upregulate DNA methyltransferases DNMT1, DNMT3A, and DNMT3B to promote genomic instability 17, induce methylation of *CXCL14* to promote tumor growth via angiogenesis 18. Therefore, the antibody against HPV16 E7 proteins could be a valuable tool for better understands the crucial role of HPV16 E7 protein in carcinogenesis both experimentally and clinically.

There are various methods to detect HPV16 E7 protein, from detecting antibodies to HPV16 E7 protein in serum by radioimmunoprecipitation assay 19, ELISA 20, immunoassay platform 21 or fluorescence detection platform 22, to directly detect HPV16 E7 protein by immunohistochemistry study via polyclonal 23 or monoclonal 24 antibody. Compared to indirect antibodies detection, the immunohistochemistry study could directly and visually demonstrate HPV16 E7 oncoproteins are present. The increasing positive coincidence rate with malignancy grade and 100% negative coincidence rate between HPV genotyping and immunohistochemistry also indicate that the immunohistochemistry study is more specific in detecting persistent infection rather than transient infection, especially in early cervical cancer. Besides, it could prevent excess diagnosis or therapy to reduce the public health burden. Compare to monoclonal antibodies, polyclonal antibodies were considered a higher sensitivity in the application and less procedure in preparation. Furthermore, immunohistochemistry studies with lower costs and easier operation are especially fit for the transitioning economies without sufficient skilled pathologists. In this study, we have prepared a repeatable, accessible, productive, sensitive and low-cost anti-HPV16 E7 polyclonal antibody.

Previous studies indicate that both HPV16 E7 mRNA 25 and protein 26 expression is higher in women with HISL than LISL, a severe cervical dysplasia than a slight cervical dysplasia. It is similar to our result in comparing the HPV16 E7 protein-positive rate with histology and cytological malignancy grades indicated that HPV16 E7 oncoprotein could be a useful biomarker for identifying persistent HPV infection from malignant and pre-malignant lesions. In summary, these advantages noted that our homemade HPV16 E7 polyclonal antibody could be a valuable tool to evaluate the severity of cervical lesions clinically with higher specificity, higher sensitivity, visuality, more economically and easier operation, especially in low/medium HDI regions. However, in our study, there is no relationship between HPV16 E7 protein expression levels and tumor sizes, tumor differentiation, lymph node metastasis, FIGO stage or lymphovascular space invasion. It might contribute to a variety of confounding biases and suggest the HPV16 E7 protein plays a crucial role in tumorigenesis rather than a prognosis marker. For the limitation, our study applied the antibody on cervical tissue specimens rather than cytological samples make it hard to evaluate its diagnosis role. Further research focuses on early screening or diagnosis could better illustrate the value of our polyclonal antibody.

In conclusion, the present study demonstrates the preparation of HPV16 E7 polyclonal antibody and a reliable immunohistochemistry protocol for its application in various cervical lesions. The positive coincidence rate of HPV16 E7 protein immunohistochemistry stain compared to HPV genotyping analysis in CIN1, CIN2, CIN3, and cervical cancer was 15%, 93%, 100%, and 100%, while the negative coincidence rate was 100% accordingly. It revealed an elevated HPV16 E7 protein-positive rate in HPV16 DNA-positive cervical lesions with increasing histological and cytological malignancy grades. However, there is no relationship between HPV16 E7 protein expression levels and tumor sizes, tumor differentiation, lymph node metastasis, FIGO stage or lymphovascular space invasion in cervical cancer. The preparation of the HPV16 E7 protein *in vitro* may provide a basis for further research focusing on the role of HPV E7 proteins in various cancers.

## Figures and Tables

**Table 1:** HPV16 E7 protein expression in various cervical tissue specimens.

Histology	HPV16 E7 protein expression levels, N				Total, N	Positive Rate (%)
	Negative	Low	Moderate	High		
Chronic cervicitis	33	0	0	0	33	0
Cervical Intraepithelial Neoplasia 1 (CIN 1)	34	5	1	0	40	15
Cervical Intraepithelial Neoplasia 2 (CIN 2)	3	2	10	28	43	93
Cervical Intraepithelial Neoplasia 3 (CIN 3)	0	1	2	35	38	100
Cervical cancer	0	0	2	59	61	100

HPV16 E7 protein expression levels were classified into four groups (negative, low, moderate, and high) with relative staining levels.

**Table 2:** HPV16 E7 protein expression in various cervical cytological specimens.

Cytology	HPV16 E7 protein-positive, N		Total, N	Positive Rate (%)
	Negative	Positive		
Negative for Intraepithelial Lesion or Malignancy (NILM)	41	20	61	67.2
Atypical Squamous Cells of Undetermined Significance (ASC-US)	21	8	29	72.4
Low-grade Squamous Intraepithelial Lesion (LSIL)	14	3	17	82.4
Atypical Squamous Cells, which cannot exclude a High-grade lesion (ASC-H)	20	2	22	90.9
High-grade Squamous Intraepithelial Lesion (HSIL)	49	4	53	92.5

**Table 3:** HPV16 E7 protein expression in cervical cancer.

Variables	Total	Moderate HPV16 E7 protein expression, N	High HPV16 E7 protein expression, N	*P* value
Tumor size, cm				
< 4	58	2	56	r=0.042, *p*>0.05
≥ 4	3	0	3	
Degree of differentiation				
Well	19	0	19	r=0.124, *p*>0.05
Moderate and poor	42	2	40	
Lymph node metastasis				
N0	54	2	52	r=0.066, *p*>0.05
N1	7	0	7	
FIGO stage				
Ⅰ and Ⅱ	54	2	52	r=0.066, *p*>0.05
Ⅲ	7	0	7	
Lymphovascular space invasion (LVSI)				
Negative	54	2	52	r=0.066, *p*>0.05
Positive	7	0	7	
